# Mycobacterium abscessus Prosthetic Joint Infections of the Knee

**DOI:** 10.7150/jbji.36286

**Published:** 2019-09-25

**Authors:** Lydie Nengue, Mark Anthony A. Diaz, Courtney E. Sherman, Arveen Bhasin, Claudia R. Libertin

**Affiliations:** 1 Division of Infectious Diseases, Mayo Clinic, Jacksonville, Florida, USA; 2 Department of Orthopedic Surgery, Mayo Clinic, Jacksonville, Florida, USA; 3 Division of Allergy, Mayo Clinic, Jacksonville, Florida, USA

**Keywords:** *Mycobacterium abscessus*, Prosthetic Joint Infections, Knee Arthroplasty, Rapidly Growing Mycobacteria, Non-tuberculous mycobacterium.

## Abstract

*M. abscessus* complex prosthetic joint infections (PJI) of the knee are rare. We present a patient with *an M. abscessus subsp. massiliense*, a nontuberculous mycobacterium (NTM), peri-prosthetic knee infection who presented with wound drainage followed by sepsis. The published peer-reviewed literature on knee PJIs due to this organism is reviewed to highlight its clinical presentation,symptomatology, microbiology, surgical interventions, antimicrobial regimens, and outcomes.

## Introduction

*M. abscessus* subsp.* massiliense* is a member of the Rapidly Growing Mycobacteria (RGM). RGM-infections propose a diagnostic and therapeutic challenge to clinicians because of the organism's growth rate, lack of isolation on routine bacterial cultures, and unique resistance to most traditional anti-tuberculous agents. Post-traumatic non-healing wound infections with poor response to broad-spectrum antibiotics should alert physicians to consider the presence of a non-tuberculous mycobacterial (NTM) disease. The backbone therapy, if susceptible, is a macrolide antibiotic. Infected hardware removal is essential for cure. Patients should also be aware of the complexity of the treatment course that includes prolonged antimicrobial therapy and many associated adverse drug reactions.

## Case Presentation

An 82-year-old Caucasian male with degenerative joint disease and hypertension presented with progressive right knee pain. He underwent a right total knee arthroplasty (TKA) after failed multiple trials of local steroid injections to alleviate pain and swelling. His surgery was complicated by poor wound healing, joint swelling, and drainage from the surgical site as early as the first week post-TKA. He developed a fever higher than 102^°^F, hypotension of 90/62mm Hg, and syncope after 7 weeks post-TKA requiring emergent hospitalization and fluid resuscitation. Vancomycin (1 g every 12 hours IV) and cefepime (2 g every 8 hours IV) were empirically started. Debridement and implant retention (DAIR) for the infected TKA occurred. After a week, 3 out of 3 intraoperative synovial fluid cultures yielded *Mycobacterium abscessus* subsp*. massiliense*. Susceptibilities were sent to the National Jewish Health in Denver, Colorado (Table 1, Isolate #1). A two-drug combination with Azithromycin 500 mg oral daily and Cefoxitin 3 g every 6 hours IV was started. Cefoxitin was discontinued after two weeks due to drug rash; Tigecycline (50 mg IV daily after 100 mg loading dose) replaced Cefoxitin. He was maintained on the two-drug regimen (Azithromycin and Tigecycline) for approximately three months after which he was placed on chronic suppressive therapy of oral Ciprofloxacin 750 mg once daily.

**Figure 1:**
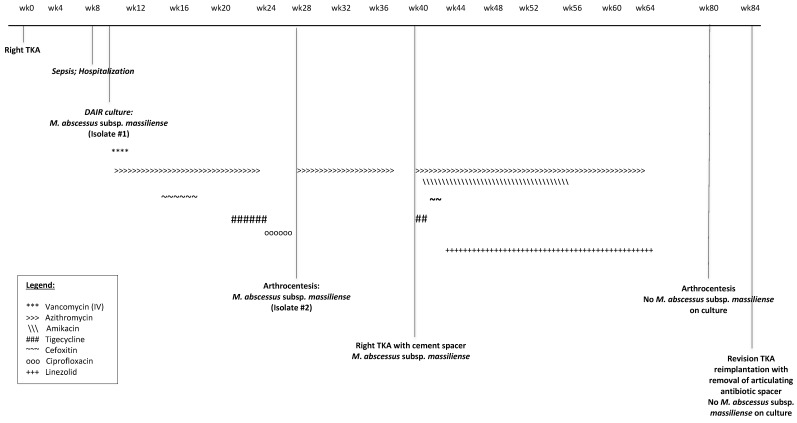
Timeline of Management and Interventions for *M. abscessus* subsp. *massiliense* Knee PJI at Both Institutions

Due to persistent severe knee pain, he self-referred to our institution for an infectious diseases consultation. At that time, all antibiotics were stopped. Allergology service was consulted to determine if Cefoxitin re-challenge would be an option, and surgery was consulted for a diagnostic arthrocentesis. *M. abscessus* subsp.* massiliense* was isolated after 19 days on BBL Mycobacteria Growth Indicator Tube and Remel Middlebrook 7H11/Mitchison 7H11 Selective Biplate agar. The isolate was identified as *Mycobacterium abscessus* group using MALD-TOF platform (Table 1, Isolate #2). He declined immediate two-stage revision for the infected TKA, but opted to take oral Azithromycin (500 mg daily orally) suppressive monotherapy to attend a major life event. (Figure 1)

After 2 months on Azithromycin, he underwent the initial part of the two-stage revision arthroplasty beginning with hardware removal and placement of an antibiotic impregnated static cement spacer (10 g of amikacin per Simplex P 80 grams). Intraoperative cultures yielded the same organism. (Table 1, Isolate #3) An audiogram done before initiating treatment documented a baseline sensorineural hearing loss, which never worsened during his 8 month antimicrobial therapy. A three-drug regimen consisted of 2 parenteral antibiotics (Tigecycline 50 mg daily and Amikacin 25 mg/kg three times weekly) with oral Azithromycin 500 mg daily. Gastrointestinal symptoms and syncope limited tigecycline use while Cefoxitin was complicated by the recurrence of rash and resistance on susceptibilities. Linezolid 600 mg oral twice daily with dosing adjustments made to as low as 300 mg daily to keep platelet levels above > 100,000 per microliter replaced Cefoxitin. Linezolid (300 mg oral daily) and Azithromycin (500 mg oral daily) were continued for two more months to complete a total duration of eight months of antimicrobial therapy post-hardware removal. Complete blood count, creatinine, liver function test, sedimentation, C-Reactive protein and amikacin drug levels (peaks/troughs) were monitored twice weekly with periodic audiometry testing throughout the duration of therapy.

Repeat arthrocentesis and cultures for bacteria, fungi, and mycobacteria done after three months of completion of all antimicrobials yielded no growth. Follow-up of ESR and CRP showed a greater than 5-fold decline from original values. Four sets of intra-operative cultures during TKA re-implantation a month after the repeat arthrocentesis were negative for the NTM-infection (NTMI). There was no evidence of microbiological and clinical relapse of the NTMI after more than 6-months of follow-up.

## Discussion

NTM species are ubiquitous organisms found in the environment. The source of the bacterium was not identified in this case, but the two possible sources of infection are his prior corticosteroid injections or initial TKA surgery. The use of contaminated injections inoculation during the process of mixing the steroids, or upon the injection procedure have been reported sources of joint and cutaneous infections for NTMI.^1^ Contamination during the procedure or early post-op soilage of surgical field, application of infected prosthesis, use of contaminated water, or shedding from transient NTM colonization can all be other causes of an NTMI.^2^ In an investigation of an outbreak of NTM PJI in Oregon from 2010 - 2016, infections were even associated with the presence of a surgical instrument representative present in the operating room during the procedures.^3^

Review of the literature 4-9 yields a total of 7 additional cases of *M. abscessus* PJI of the knee (Table S1). The clinical presentations of RGM PJIs are similar to musculoskeletal NTMI of the lower extremities where subacute arthritis may progress to osteomyelitis.^10^ All 8 cases (Table S1 in Supplementary Material) were greater than 60 years of age and female (median age 71 years; range 48-83 years) except case 8. All patients were normal hosts. The time interval between the prosthesis implantation and the onset of symptoms varied tremendously, ranging from days to 3 years (median = 24 weeks). As reported by Diaz et al^10^., there is a gap in the interval between symptom presentation and diagnosis of NTMI in lower extremities that was evident also with PJIs. The main reasons for the diagnostic delay were nonspecific characteristics of infection, lack of familiarity with NTMI, and lack of clinical suspicion of NTMI until the infection does not resolve after antimicrobials. Two cases are early onset PJIs, and five are delayed PJI of the knee. One patient died in hospice. Of note, cases before 2013 may not be counted in this review based on changes made to the classification and nomenclature of the *M. abscessus* complex between 1992 and 2013. Previously, *M*. *abscessus* and *M. chelonae* were considered a single specie, but *M. abscessus* was reclassified as an individual specie in 2002.^10^ The distinction between closely related species such as *M. chelonae* and *M. abscessus* complex relied on phenotypic differences, which were few. Consequently, the number of cases before 2013 may be impacted by nomenclature changes.

Although unusual, septic arthritis with the development of concurrent systemic sepsis has been associated with poor outcomes in terms of infection recurrence and arthritis progression.^11^ Debridements of TKA without hardware removal can lead to treatment failure and longer durations of therapy. Antimicrobial t*reatment regimens are complex and directed by susceptibility data, when available. Of note, M. abscessus* complex is typically resistant to most antimicrobial agents. The Clinical and Laboratory Standards Institute recommends testing RGM for susceptibility to various antimicrobials such as those listed in Table 1. Amikacin, Cefoxitin, and Clarithromycin have the best *in-vitro* antimycobacterial activity and are commonly used in the cases. Macrolides are the cornerstone of treatment and were used throughout the eight months of therapy (Figure 1) in our case. Isolates of *M. abscessus* subsp. *abscessus* may have resistant MICs after extended incubation, which is the result of an inducible methylase gene *erm*(41) which confers macrolide resistance.^12^ Therefore, testing of clinically significant isolates of RGM for *erm*(41) gene inducibility for 14 days for Clarithromycin at a reference laboratory is indicated. Clofazimine is a second-line alternative used for MDR-TB (Multi Drug Resistant-Tuberculosis) and other NTMI. None of the PJI cases used clofazimine. Novartis no longer has an Expanded Access Program for NTM infections.

The recommended duration of therapy for osteoarticular NTMI is 6 to 12 months.^10^ Table S1 notes the various lengths of combination antimicrobials given for *M. abscess*us knee PJIs. Our case demonstrates that post-TKA-resection, 8 months of therapy was curative. A two-staged orthopedic procedure combined with negative cultures following a 2-month antimicrobial holiday is advised before re-implantation. With hardware removal and prolonged antimicrobial therapy, all cases clinically and microbiologically were cured of *M. abscessus* PJI.

In conclusion, NTM PJI is an emerging infectious disease. Diagnosis should be suspected especially in non-healing infections poorly responsive to broad spectrum antibiotics. Patients and clinicians should be aware that early, aggressive and prolonged interventions are keys to treatment. By the same token, clinical and drug monitoring are essential to compliance and completion of the regimen.

## Supplementary Material

Supplementary Table S1.

## Figures and Tables

**Table 1:** *Mycobacterium abscessus* subsp. *massiliense* susceptibility reports

Antibiotic	Isolate #1: MIC (ug/mL)^1^	Interpretation	Isolate #2: MIC (ug/mL)²	Interpretation	Isolate #3: MIC (ug/mL) ^2^	Interpretation
Cefoxitin	32	I	64	I	128	R
Imipenem	8	I	32	R	64	R
Ciprofloxacin	4	R	>4	R	>4	R
Moxifloxacin	>4	R	8	R	>8	R
Clarithromycin	0.5	S	0.25	S	0.5	S
Amikacin	16	S	16	S	16	S
Tobramycin	>16		>16	R	>16	R
Doxycycline	>16	R	>16	R	>16	R
Minocycline	Not tested		>8	R	>8	R
Tigecycline	1	S	0.12		0.25	
TMP/SMX	>4/76	R	8/152	R	>8/152	R
Linezolid	>16	R	16	I	8	S
Azithromycin	≤16	S	Not tested		Not tested	
Clofazimine	≤0.5	S	Not tested		Not tested	

Isolate #1: Isolate from incision and drainage of soft tissue and bone with polyethylene exchange of right knee done 2 months after original TKA Isolate #2: Isolate from arthrocentesis Isolate #3: Isolate cultured from the right knee
