# Supplementary material for: Mycobacterium abscessus Prosthetic Joint Infections of the Knee
Source: J Bone Jt Infect. 2019 Sep 25;4(5):223–6. doi: 10.7150/jbji.36286 (PMC6831809; doi:10.7150/jbji.36286)
Supplement: Supplementary file 1 — Supplementary Table S1. [file jbjiv04p0223s1.pdf]

Table S1. Summary of Clinical Characteristics and Outcomes of Patients with *M. abscessus* PJI of the Knee

Patient	Publication Date	Age (years)/ Sex	Underlying Disease	Symptom(s)	Time to Onset of Symptoms Post-operatively [a] (weeks)	Year of Implantation	Organism-subspecies/ Method of Identification/Number of Positive Samples	Surgical intervention	*M. abscessus* Therapy	Total Duration of Therapy (months)	Duration of Follow-up (weeks)	Outcome	Antibiotic Cement Spacer Used
1	1995	48/F	Chronic hepatitis	Pain in hip and knee	144	Not documented	*M. abscessus* / NK / NK	Removal of hip prosthesis with removal of the tobramycin impregnated beads from the hip	AMK+ERY+CIP<br>CFX + CLR	10	60	Cured	Not done
2	2007	71/F	Bronchiectasis colonized by *M.abscessus;* rheumatoid arthritis (immunosuppressed)	Pain; swelling of knee	Not documented	1979 (knee)<br>1977 (elbow)	*M. abscessus*/ NK / 3 of 6	Resection arthroplasty	CFX + CLR	0.5	3	Palliative care	Not documented
3	2011	72/F	Osteoarthritis	Fever; pain; swelling of the knee	20	Not documented	*M. abscessus* / NK / NK	Index right TKA<br>Removal of the prosthesis (resection arthroplasty) + synovectomy with insertion of a spacer<br>Re-implantation of a prosthetic joint	CFX+AMK+CLR<br>DOX+CIP+CLR+ AMK	10	40	Cured	Not documented
4	2017	83/F	None	Erythema; swelling of the knee	3	Not documented	M. abscessus / NK / 4 of 11	Open debridement with polyethylene insert exchange<br>Open debridement with removal of prostheses and vancomycin loaded cement spacer insertion<br>Open debridement with insertion of vancomycin loaded articulating cement spacers<br>Repeated surgical debridement and insertion of azithromycin loaded articulating cement spacers<br>Revision TKA<br>Open debridement with polyethylene insert exchange	CFX +AZM<br>AMK + CFX+MOX+CLR<br>AMK+CFX+AZM<br>AZM+ RIF +CIP<br>CIP+RIF	20	204	Cured	Vancomycin (4g per 40g batch of cement)<br>Azithromycin loaded cement (2g per 40g batch of cement) used post-surgical debridement + Azithromycin-loaded cement (one gram per 40 g batch of cement) used for implant fixation.
5	2017	71/F	None	Not reported	52	Not documented	*M. abscessus* / NK / 2 of 2	Index primary TKA on Right knee<br>Staged revision arthroplasty due to PJI 1month post primary TKA<br>Open debridement with retention of the prostheses<br>Open debridement with removal of the prostheses and insertion of antibiotic cement spacers<br>Revision TKA<br>Additional open debridement (due to relapse post revision TKA)<br>Open debridement with insertion of articulating cement spacers loaded with Antibiotics<br>Repeated open debridement with insertion of cement spacers loaded with antibiotics<br>Reimplantation arthroplasty using varus–valgus constrained-type prostheses with antibiotic-loaded cement	CLR + LEV+ RIF<br>AMK + CFX + CLR<br>AMK+ TGC+ CLR<br>AMK+CLR<br>AMK+TGC+CLR<br>CIP + CLR	12	96	Cured	Tigecycline 4g + Clarithromycin 4g both per 40g batch of cement

6	2017	71/F	Hypertension	Abscess (tender swollen knee joint with drainage)	96	Not documented	*M. abscessus* / Intraoperative specimens & synovial fluid aspirate on BACTEC MGIT 960 system / NK	Index primary TKA on the right knee:<br>Resection arthroplasty (extraction of loose implants and their replacement with gentamicin impregnated cement spacer)<br>Re-debridement with change of cement spacer<br>Revision TKA with long stemmed tibial & femoral components	CLR+ LEV+ AMK<br>CLR + LEV + IMP	5	96	Cured	Gentamicin (dose not documented)
7	2018	61/F	Baker's cyst	Knee pain; swelling	144	Not documented	*M. abscessus* subsp. massiliense/ NK / NK	Two stage antibiotic spacer explantation and exchange with debridement of the bone, complete synovectomy and removal of all implants<br>Open biopsy<br>Replantation TKA	AMK+CFX+TGC<br>AMK + CFX + CLR	5	192	Cured	Amikacin 10g + Cefoxitin 24g<br>Tobramycin (dose not documented)
8 (Present case)	2019	83/M	Degenerative joint diseases; hypertension	Drainage; joint warmth; pain and swelling of knee.	8	2017	*M. abscessus* subsp. *massiliense* / subsp. *massiliense* identified by gel analysis for erm (41) gene product and sequence analysis for hsp65 gene on synovial fluid aspirates and intraoperative cultures / 3 of 3	Debridement and irrigation of soft tissue and bone with polyethylene exchange with retention of the hardware.<br>Revised right TKA with articulated spacer placement arthrocentesis (2 stage exchange)<br>Removal of articulating antibiotic spacer and revision right TKA reimplantation	AZM+CFX+TGC<br>AZM+TGC+CIP<br>AZM (suppression)<br>AZM+AMK+TGC<br>AZM+CFX+AMK<br>AZM+AMK+LNZ<br>AZM+LNZ	10	24	Cured	Amikacin 10g

[a] Interval between last surgical procedure (prosthesis implantation) and the first appearance of symptoms

F: Female

M: Male

NK: Not Known

TKA: Total Knee Arthroplasty

PJI: Prosthetic Joint Infection
